# Associations of childhood diet quality scores with arterial stiffness and carotid artery intima-media thickness in adolescence/early adulthood: findings from the ALSPAC cohort

**DOI:** 10.1017/S0007114523002763

**Published:** 2024-02-28

**Authors:** Genevieve Buckland, Kate Northstone, Pauline M. Emmett, Caroline M. Taylor

**Affiliations:** 1 Centre for Academic Child Health, Bristol Medical School, University of Bristol, Bristol, UK; 2 Department of Population Health Sciences, Bristol Medical School, University of Bristol, Bristol, UK

**Keywords:** Diet quality scores, Arterial function, Pulse wave velocity, Carotid intima-media thickness, Children and adolescents, Avon Longitudinal Study of Parents and Children (ALSPAC), Prospective cohort study

## Abstract

This study examined the relationship between childhood diet quality and arterial stiffness and thickness during adolescence/early adulthood. Participants were from the Avon Longitudinal Study of Parents and Children (ALSPAC) with dietary data at ages 7, 10 and 13 years and pulse wave velocity (PWV) and carotid intima-media thickness (cIMT) at ages 17 and/or 24 years. Diet quality (DQ) was assessed using five scores: a children’s Mediterranean-style diet (C-rMED) *Z*-score, a children’s Dietary Inflammatory *Z*-score (C-DIS), a DASH diet *Z*-score, a children’s Eatwell Guide (C-EWG) *Z*-score reflecting UK dietary guidelines and a data-driven obesogenic *Z*-score. Adjusted regression models examined the associations between DQ scores at 7–13 years and PWV and cIMT at 17 and 24 years. In adjusted models, a high *v*. low Obesogenic *Z*-score at 7 and 10 years was associated with higher PWV at 17: *β* 0.07 (95 % CI 0.01, 0.13) and *β* 0.10 (95 % CI 0.04, 0.16), respectively. A high *v*. low C-rMED *Z*-score at 7 years was associated with lower PWV at 17 (*β* −0.07; 95 % CI −0.14, −0.01). A high (more anti-inflammatory) vs low C-DIS *Z*-score at 10 years was associated with a lower PWV at 17 years: *β* −0.06 (95 % CI −0.12, −0.01). No other associations were observed. In conclusion, an Obesogenic dietary pattern in childhood (7–10 years) was related to increased arterial stiffness, while Mediterranean-style and anti-inflammatory diets were related to decreased arterial stiffness in adolescence. This highlights the importance of establishing healthy dietary habits early in life to protect against vascular damage.

Cardiovascular disease (CVD) remains the leading cause of death globally^(1)^. In the UK, heart and circulatory diseases contribute to a quarter of all deaths, and almost of a third of these are classed as premature^(2)^. Although CVD is normally diagnosed from middle age, the pathophysiological processes leading to CVD can start in early life due to an interplay of environmental and genetic factors^(3)^.

Early subclinical cardiovascular alterations include changes to the functional and structural properties of the arteries. For example, arterial stiffness (loss of elasticity) is an important early marker of vascular functional damage and is partly caused by a loss of elastic fibres and stiffer collagen fibres in the arterial wall and is also linked to inflammation and hypertension^(4)^. It can be measured non-invasively using pulse wave velocity (PWV: the speed at which a blood pressure pulse travels between two points in the same artery)^(5,6)^. Structural damage to large arteries can be caused when deposits of cholesterol and its esters (fatty streaks) inside the arteries develop into plaques in a condition termed atherosclerosis. Increased carotid intima-media thickness (cIMT) is a surrogate marker of early pre-clinical atherosclerosis and can be assessed using non-invasive high-resolution ultrasound to measure the distance between the intima layer (innermost layer of the artery wall) and the subsequent layer – the media.

Increased arterial stiffness (measured by PWV) and arterial wall thickness (measured by cIMT) are pre-clinical outcomes that are strong independent predictors of incident CVD and mortality^(4,7–9)^. These markers of early vascular dysfunction are useful tools for detecting future increased CVD risk in adolescents and young adults well before overt clinical manifestations occur. Identifying modifiable risk factors which are associated with arterial stiffness and thickness in early life is therefore key, as they may contribute to, and be used to ameliorate, the risk of developing CVD later in life.

CVD has been linked to a life-course accumulative exposure to unhealthy diets, and there is now a substantial line of evidence in adults showing that dietary habits are associated with cIMT and PWV, as intermediate CVD risk markers^(10–14)^. For instance, a Western dietary pattern (DP) identified using data-driven methods in a prospective cohort study in the USA was positively associated with increased cIMT in women in midlife^(12)^. Research within cohort studies in France have found that a nutritionally poor DP (characterised by high meat and alcohol consumption or low intakes of fruity desserts, vegetables and legumes) were related to an increased stiffening of large arteries, shown by higher PWV^(11,15)^. In a European randomised control trial, a 1-year Mediterranean-style dietary intervention in 65–79-year-olds significantly decreased the augmentation index, another measure of arterial stiffness^(16)^.

Two established prospective studies, the Young Finns Study and Amsterdam Growth and Health Longitudinal Study Research, have examined how dietary habits are related to vascular damage in younger populations. They have both reported that healthy nutrients (MUFA and *n*-3 PUFA and fibre), certain foods (fruit and vegetables) and a Mediterranean DP consumed throughout childhood were associated with favourable effects on arterial stiffness and cIMT in adulthood^(17–21)^, while a traditional Finnish DP (rich in potatoes, butter, sausages, rye, milk and coffee) was related to increased arterial stiffness^(22)^. Other epidemiological studies in children exploring the relationship between DP and markers of vascular dysfunction are mostly restricted to higher risk paediatric populations, such as children with obesity, type 1 diabetes mellitus, or familial hypercholesterolaemia, and have produced mixed findings^(23–27)^.

Previous research within the Avon Longitudinal Study of Parents and Children (ALSPAC) has shown that several renowned *a priori* DP (a Mediterranean diet, an anti-inflammatory diet, and the Dietary Approaches to Stop Hypertension (DASH) diet, as well as a children’s Eatwell Guide (C-EWG) score reflecting adherence to UK dietary guidelines) and an Obesogenic-DP, derived using reduced rank regression (RRR) in childhood, were all prospectively associated with composite cardiometabolic risk score at 17 and/or 24 years^(28–31)^. It is also of scientific interest to understand which of these major DP are more predictive of early markers of vascular dysfunction, as the distinguishing features of a certain DP may be useful to highlight in preventative strategies. Thus, the purpose of this study was to prospectively explore the association between diet quality (DQ) throughout childhood, assessed using five distinct DQ indices, and two measures of vascular function: arterial stiffness (measured by PWV) and arterial wall thickness (measured by cIMT), in adolescence and early adulthood.

## Methods

### Cohort description

The study is based on the index children of ALSPAC – a birth cohort study set-up in the 1990s to investigate how genetic and environmental characteristics influence the health and development of children across the life course^(32)^. Full details of the study are available on the ALSPAC website (www.alspac.bris.ac.uk) and have been published previously^(33–35)^. The study initially enrolled 14 541 eligible pregnant women from the South West of England with expected due dates between 1991 and 1992, which resulted in 13 988 children alive at 1 year. Subsequent recruitment phases in 1999 (child mean age: 7·5 years) and in 1999–2012 (child mean age: 17·8 years) resulted in a final sample of 14 901 eligible children alive at 1 year of age^(35)^. At birth, the ALSPAC families were relatively representative of the population in the area^(33)^. Extensive data have been collected from the parents and their children during periodic follow-ups since recruitment, using a combination of questionnaires, medical records and in-person clinical visits. Study data were collected and managed using Research Electronic Data Capture (REDCap) electronic data capture tools hosted at the University of Bristol^(36)^. REDCap is a secure, web-based software platform designed to support data capture for research studies. The study website contains details of all the data that are available through a fully searchable data dictionary and variable search tool (http://www.bristol.ac.uk/alspac/researchers/access).

### Ethical approval and informed consent

Ethical approval for the study was obtained from the ALSPAC Ethics and Law Committee and the Local Research Ethics Committee (http://www.bristol.ac.uk/alspac/researchers/research-ethics/), and the study conformed to the guidelines within the Declaration of Helsinki. Consent for biological samples were collected in accordance with the Human Tissue Act (2004). Written or verbal informed consent for the use of data collected via questionnaires and clinics was obtained from participants following the recommendations of the ALSPAC Ethics and Law Committee at the time. At all the clinics, the children were invited to give consent, when appropriate. Full details of the ALSPAC consent procedures are available on the study website (http://www.bristol.ac.uk/alspac/researchers/research-ethics/).

### Dietary assessment

**Fig. 1.**
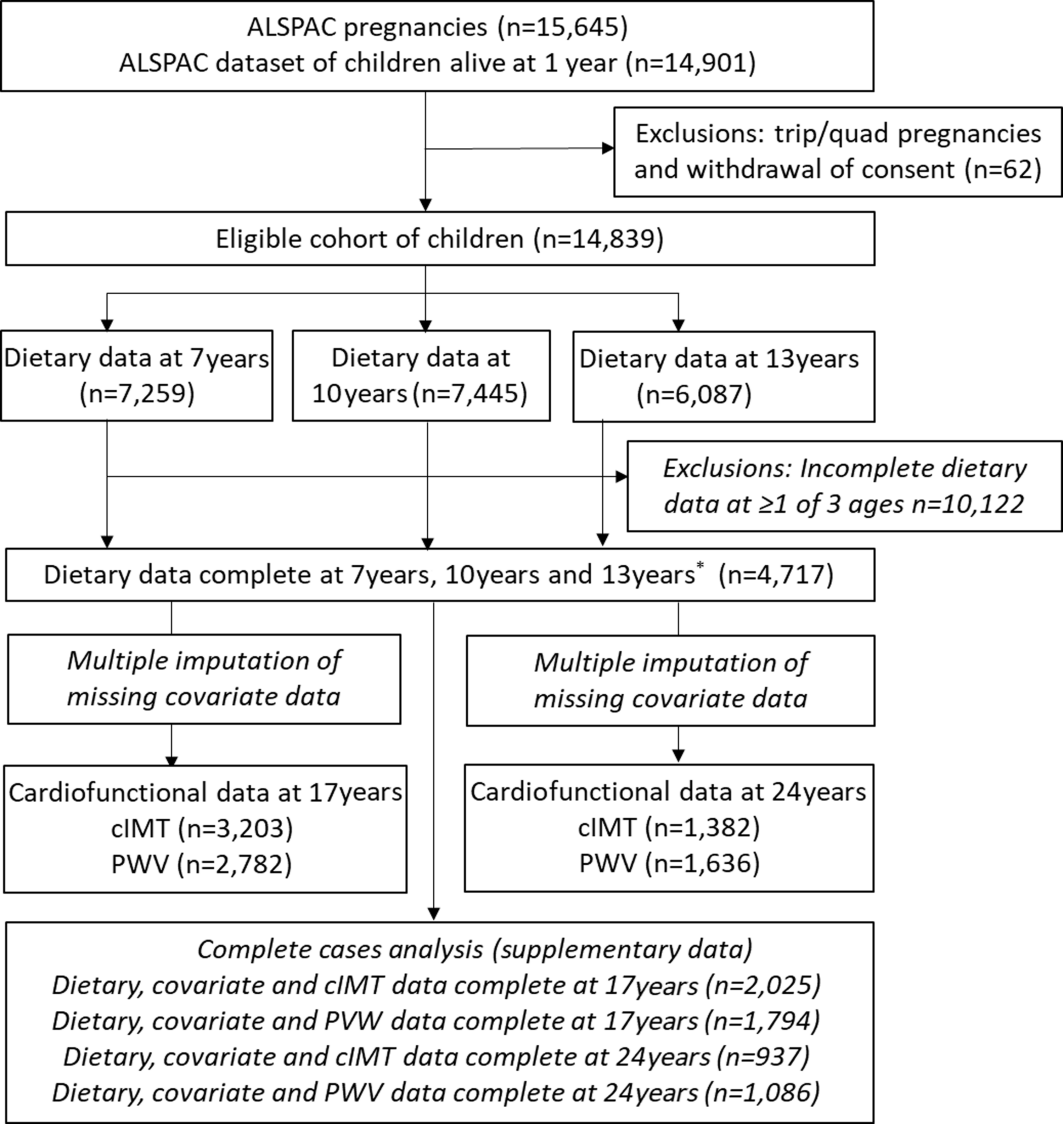
Study flow diagram for participant data from the Avon Longitudinal Study of Parents and Children (ALSPAC). The present study uses data from participants with complete dietary data at 7, 10 and 13 years and complete data on the cardiofunctional data at 17 years and 24 years and uses multiple imputation for missing data in covariates. *Complete dietary data refers to at least one diet diary recorded for a child at all three ages (7, 10 and 13 years). Three complete days of diet diary data were available for 86·5 %, 83·6 % and 78·4 % of children at 7, 10 and 13 years, respectively. cIMT, carotid intima-media thickness; PWV, pulse wave velocity.

Dietary intake was assessed at 7, 10 and 13 years using data from 3-d diet diaries; the full details have been described previously^(37)^. The diet diaries were completed by the parents/caregivers when the children were 7 years of age and by the children with support from the caregiver when the children were 10 and 13 years of age. All food and drink consumed by the child over 2 weekdays and 1 weekend day were recorded. Details of foods and drinks consumed were noted using standard household measures and included a full description of the food and the amount offered, with a separate section for additional details including leftovers. A nutritionist then reviewed the diaries with the children/caregiver to check for completeness or discrepancies and to clarify portion sizes. This was done during clinical assessment visits; the mean age at attendance was 7·5 years (sd = 0·3), 10·6 years (sd = 0·2) and 13·8 years (sd = 0·2). Food and drinks were converted into weights using household measures or standard portion sizes relative to the age of the child. The diet diary data were coded and linked to food composition tables using DIDO (Diet In Data Out). McCance and Widdowson’s British food composition tables were used to calculate nutrient intakes^(38)^. The mean weight (g) of each food group consumed over the 3 d was used. Plausibility of dietary reporting was calculated using an individualised method based on the ratio of energy intake to estimated energy requirement and its 95 % CI^(39,40)^ and is explained in greater detail elsewhere^(30)^. Data from dietary diaries were available for 7259 children at 7 years, for 7445 at 10 years and for 6087 at 13 years. 4717 children had complete dietary data at all three ages (Fig. 1).

### Exposure variables: dietary quality scores

Five distinct DQ scores were constructed for each participant based on the dietary data that were available at all three ages (7, 10 and 13 years old). The DQ scores included the children’s relative Mediterranean-style diet (C-rMED) score^(28)^, a DASH Diet Score^(41)^, a children’s Dietary Inflammatory Score (C-DIS)^(29)^, an C-EWG score^(30,42)^ and an Obesogenic-DP score^(43)^. All of the scores were converted to *Z*-scores to standardise the units and scales. These DQ scores have been analysed previously in the ALSPAC children, and the full methodological details of how they are constructed are published elsewhere^(28–30,41,43)^ and described in supplementary material methods section and in Table 1. For all of the DQ scores, except the Obesogenic-DP score, higher scores reflect ‘healthier’ DP. In contrast, for the Obesogenic-DP score, higher scores indicate a more ‘unhealthy’ DP, characterised by high energy density, high free sugars and fat, and low fibre density^(43)^.

**Table 1.** Methodological details of the five diet quality scores analysed

	Diet quality score	Purpose	No. foods/nutrients	Foods/nutrients included	Scoring method	Energy density method used*
1	Children’s relative Mediterranean-style diet (C-rMED)^(28)^	Reflects alignment to a Mediterranean-style dietary pattern, relative to the study population	8	MD-style components: fruit, vegetables, pulses, cereals and cereal products, fish/seafood, dairy products, and a lipid ratio (sum of MUFA and PUFA divided by SFA g/d). Non-MD-style components: meat and meat products	Tertiles of intake scored from 0 to 2 for MD-style components and scoring is reversed for non-MD components	Yes
2	Children’s Dietary Inflammatory Score (C-DIS)^(29)^	Estimates the overall inflammatory potential of the diet	24	Energy, carbohydrate, protein, total fat, saturated fat, monounsaturated fat, polyunsaturated fat, dietary cholesterol, fibre, vitamin A, vitamin B_6_, vitamin B_12_, vitamin C, vitamin D, vitamin E, folic acid, *β*-carotene, thiamine, riboflavin, niacin, Fe, Mg, Zn and Se	Each food/nutrient is: (1) divided by total energy; (2) standardised using *Z*-scores; and (3) multiplied by its corresponding positive or negative inflammatory weight (previously defined)	Yes
3	Dietary Approaches to Stop Hypertension (DASH) diet score^(41)^	Reflects alignment to DASH-style dietary pattern, originally aimed to reduce hypertension.	8	Healthy components: fruit, vegetables, nuts and legumes, wholegrains, low-fat dairy products and unhealthy components: red and processed meat, Na and non-milk extrinsic sugars	Quintiles of intake scored from 1 to 5 for healthy components, and scoring is reversed for unhealthy components	Yes
4	Children’s Eatwell Guide (C-EWG) score^(30)^	Reflects adherence to UK government dietary recommendations, according to the Eatwell Guide.	9	Total fat, saturated fat, free sugars, fibre, salt, fruit and vegetables, non-oily fish, oily fish and red and processed meat	Adhering to guidelines: 1 point; not adhering to guidelines: 0 points	No
5	Obesogenic-dietary pattern (DP)^(31)^	Dietary pattern explains the maximum variation in response variables hypothesised to be on the pathway between food intake and obesity	Four response variables and forty-six predictor variables	Four response variables: energy density (kJ of total food energy/ total food weight (g)), percentage energy from fat, percentage energy from free sugar and dietary fibre (non-starch polysaccharide) density (g/MJ).	Data-driven dietary pattern: reduced rank regression	No

MD, Mediterranean diet.

* Energy density method: the intake of each food, nutrient or food group is calculated as a function of total energy intake (g/4184 kJ/d (g/1000 kcal/d)), before further calculations.

### Outcome variables

cIMT and PWV were measured when the participants attended the 17- and 24-year clinical visits. For cIMT, scans of the right and left common carotid arteries were taken using a high-resolution ultrasound system (13–5 MHz linear array broadband transducer: Vivid7, GE Medical), following a standardised protocol^(7)^. Full details of the procedure are detailed elsewhere^(44,45)^. In brief, participants lay on a couch with their arms by their side, while a trained clinician performed the ultrasound on both sides of their neck. Both carotid arteries were imaged longitudinally, measuring the far wall of the carotid artery over a length of 5–10 mm and 1 cm adjoining the carotid bifurcation. The left and right cIMT measurements were repeated for three consecutive end-diastolic cardiac cycles, and the mean from the left and right cIMT readings (mm) was calculated and used in the analyses. A single trained reader analysed the images.

For PWV, pressure waveforms were measured using a Vicorder device (Skidmore Medical) at carotid and femoral artery level. Full details of the procedure have been published elsewhere^(46)^. In brief, participants lay on a couch with their head raised to 30 degrees and a 100 mm wide blood pressure cuff was placed over the femoral artery in their upper right thigh and a 30 mm partial cuff over the right carotid artery in the participant’s neck. Real-time pulse wave forms were recorded simultaneously for 3 s from carotid and femoral sensor cuffs, and the pulse transit time (m/s to nearest 0·01) was computed automatically using an inbuilt cross-correlation algorithm, previously validated in adolescents^(47)^. Three sets of carotid-femoral PWV measurements were recorded within 0·5 m/s of each other, and the average was used in this analysis. The measurements were taken by one of two trained vascular technicians (independently), and the inter-observer mean difference was 0·2 m/s, sd 0·1^(48)^. A higher PWV indicates greater arterial stiffness.

### Covariate data

Covariate data were collected from the participants through parental- or self-completed questionnaires, hospital and medical records and face-to-face clinical assessment visits at baseline and during follow-up^(34)^. Data on the children’s sex, gestational age at birth and birth weight were collected by ALSPAC staff at delivery, from medical records or from birth notifications. During the clinical assessment visits, the age (months) of participants was recorded and the participants were weighed to the nearest 0·1 kg using the Tanita Body Fat Analyser weighing scale (Tanita). Height was measured using a Harpenden stadiometer (Holtain Ltd) to the nearest millimetre. BMI was calculated as weight(kg)/height(m)^2^.

Maternal data on BMI, educational attainment and social class used in this analysis were collected by self-completion postal questionnaires during pregnancy^(32)^. Pre-pregnancy BMI was calculated from self-reported height and weight collected in these questionnaires. Social class was calculated using the Office of Population Censuses and Surveys (1991) occupation-based classification, which is based on the parents’ current or last job at 32 weeks of gestation. Standardised UK social class classifications were used which ranged from social class V (lowest) to I (highest)^(49)^. Maternal and paternal social class were combined to obtain highest family social class. Maternal educational attainment was recorded as the highest qualification obtained out of Certificate of Secondary Education (CSE), vocational training, O-level/General Certificate of Secondary Education (qualifications obtained at 16 years of age), A-levels (qualification obtained at 18 years), University degree or higher.

Puberty timing was assessed using peak height velocity, previously calculated in ALSPAC using a mixed effects shape-invariant growth curve model which plots a mean growth curve using data on repeated height measurements between 5 and 20 years^(50)^. Physical activity was measured at 11 and 13 years using an Actigraph AM7164 2.2 accelerometer (Actigraph LLC), worn for 7 consecutive days around the waist at the right hip^(51)^. Participant’s data were only included in the analyses if they provided at least 3 valid days of recording; a valid day was classed as providing data for at least 10 h. Moderate-to-vigorous physical activity was calculated using the mean minutes per d in which there were >3600 accelerometer counts per minute ^(51)^. Detailed data on tobacco smoking and alcohol consumption used in this analysis were collected at 17 and 24 years via self-completed questionnaires issued during clinics or via postal questionnaires. Frequency of alcohol intake was categorised as never, monthly or less, 2 to 4 times a month, 2 to 3 times a week and 4 or more times a week. Tobacco smoking was categorised as never smoker and ever smoker which was categorised into number of cigarettes smoked in participant’s lifetime (undefined, 1–19, 20–99, and 100 or more).

### Statistical analysis

Statistical analyses were performed using Stata version 15.1 (Stata Corp.). The baseline socio-demographic and anthropometric characteristics of the 4717 participants with dietary data at all three ages were described according to tertile category of the DQ *Z*-scores at 7 years, using proportions for categorical variables and medians and interquartile range for continuous variables. Differences between categorical variables were assessed using *χ* ^2^ test and between continuous variables using Kruskal–Wallis tests due to non-parametric distribution. The representativeness of the participants included in the final analysis (compared with the initial eligible cohort) was tested by comparing their baseline characteristics to those participants excluded due to missing data on the exposure and outcome variables.

The associations between each of the five exposure variables (DQ *Z*-scores: C-rMED, DASH diet, C-DIS, C-EWG and Obesogenic-DP) and the two outcome variables (c-IMT and PWV) at 17 years and 24 years were assessed using minimally adjusted and fully adjusted multivariable regression models for each exposure and outcome combination. cIMT and PWV were analysed as continuous variables, per-unit increment. The five DQ *Z*-scores were analysed as tertiles of intake (lowest tertile as reference) and as continuous variables per sd-unit increment. The tertile groups were not completely even for the C-rMED, C-EWG and DASH score because of clustering of data values around the mean and tied values assigned to the same quantile group. Minimally adjusted models were adjusted for sex and plausibility of dietary reporting, and fully adjusted models were additionally adjusted for maternal highest education attainment, family highest social class, moderate-to-vigorous physical activity level and puberty timing. The confounders were selected *a priori* based on previous literature on factors associated with DP and cardiovascular health. Regression coefficients (*β*) with 95 % CI were presented, which estimate mean differences in PWV (m/s) or cIMT (mm) for each 1-sd increase in DQ *Z*-score or for a respective increase DQ tertile category. DQ total *Z*-score variables were also created to reflect the cumulative level of adherence to each DP over the 6-year period from 7 to 13 years. The DQ total *Z*-scores were calculated by summing together each participant’s DQ *Z*-score at 7, 10 and 13 years, separately for each of the five DP. The association between each of the five DQ total *Z*-scores (per 1-unit increment) and PWV and cIMT at 17 and 24 years was assessed using fully adjusted multivariable regression models.

Effect modification of the associations by sex was tested by including an interaction term between sex and the categorical DQ *Z*-score in regression models. As there were no significant interactions (all *P* > 0·05 using lrtest), stratified analyses by sex are not presented. In sensitivity analyses, the fully adjusted regression models were additionally adjusted for tobacco smoking status and intensity and alcohol consumption, assessed at the corresponding age each outcome was measured.

### Multiple imputation of missing covariate data

Of the participants with complete exposure and outcome data, 32–37 % had missing data on at least one of the covariates in the different fully adjusted regression models. The largest amount of missing data was for the moderate-to-vigorous physical activity variable, with 21 % at 17 years and 19 % at 24 years (online Supplementary Tables 2 and 3). Therefore, multiple imputation using chained equations (ICE command in Stata) was used to impute missing data in covariate variables. Twenty stacked datasets were generated and used in the final regression analyses, with standard combination rules for multiple imputations^(52)^. The imputation models contained all the covariates included in the final multivariable regression models and further auxiliary variables which predicted missingness of the covariates (maternal pre-pregnancy BMI, child’s BMI and total energy intake at each time of dietary data collection and Family Adversity Index^(53)^). Therefore, the basic assumption underlying multiple imputation that data were ‘missing at random’^(52)^ was met. Separate imputed datasets were created for the participants with complete dietary data and each endpoint: PWV at 17 years (*n* 2782), PWV at 24 years (*n* 1636), cIMT at 17 years (3203) and cIMT at 24 years (*n* 1382). The results from multivariable regression analyses presented in the main article are based on the imputed datasets. As part of the sensitivity analyses, the multivariable regression analyses were repeated using the complete-case datasets for PWV at 17 years (*n* 1794) and 24 years (*n* 1086) and cIMT at 17 years (*n* 2025) and 24 years (*n* 937). The distribution of covariates in the imputed and observed datasets were compared using *χ* ^2^ tests and Kruskal–Wallis tests (online Supplementary Tables 2 and 3). A *post hoc* analysis was carried out to explore the association between each of the individual food groups within the C-rMED at 7 years and PWV at 17 years (as an association was observed in the main analysis). All of the eight components of the C-rMED (as tertiles of intake) were included simultaneously in the multivariable regression models, instead of the C-rMED.

## Results

### Characteristics of study sample

From the initial eligible 14 901 participants alive at 1 year, 4717 had dietary data available at all three ages (7, 10 and 13 years) (Fig. 1). Of these participants, 3203 had data on cIMT at 17 years and 1382 had cIMT data at 24 years, while 2782 had PWV data at 17 years and 1636 had PWV data at 24 years, and these participants were included in the final analysis (Fig. 1). Table 2 describes the characteristics of the 4717 children with complete dietary data, overall and according to tertiles of the five DQ *Z*-scores calculated at 7 years old. In general, children with healthier DP (highest tertile for all DQ scores except for the Obesogenic-DP which was the lowest tertile) were more likely to be female, have mothers with a higher level of academic achievement, and with a lower BMI and come from a higher family social class. Children with higher C-DIS and C-EWG scores also had a lower mean BMI and lower total energy intake at 7 years.

**Table 2.** Characteristics of the ALSPAC children with complete dietary and outcome data, according to tertile categories of the diet quality scores

Tertiles of dietary pattern scores at 7 years	Sex			BMI at 7 years		Energy intake at 7 years		Dietary misreporting at 7 years		Maternal educational attainment		Highest household social class		Maternal pre-pregnancy BMI	
	*n* 4717			*n* 4690		*n* 4717		*n* 4689		*n* 4413		*n* 4263		*n* 4166	
Category/units	*n*	% Male	% Female	Median kg/m^2^	IQR	Median per 1000 kJ	IQR	% Mis-reporters	% Valid reporters	% CSE, vocational/O level	% A-level or above	% III, IV and V	% I and II	Median kg/m^2^	IQR
All participants		49·3	50·7	15·8	14·8–17·0	7·1	6·3–7·9	24·4	75·7	52·6	47·4	68·5	31·6	22·0	20·5–24·2
Children’s relative Mediterranean -style diet (C-rMED) score															
T1	2193	51·2	48·8	15·8	14·8–17·1	7·1	6·3–8·0	25·3	74·7	59·8	40·2	73·1	26·9	22·3	20·7–24·4
T2	1445	47·7	52·3	15·7	14·9–16·9	7·1	6·3–8·0	24·4	75·7	51·6	48·4	67·9	32·1	22·0	20·5–24·1
T3	1079	47·5	52·5	15·7	14·8–16·9	7·1	6·3–8·0	22·4	77·7	39·2	60·8	59·6	40·4	20·2	20·2–23·8
*P*-value		0·053		0·357		0·968		0·173		<0·001		<0·001		<0·001	
Dietary Approaches to Stop Hypertension (DASH) diet score															
T1	1770	51·9	48·1	15·8	14·8–17·0	7·1	6·3–8·0	26·5	73·5	63·9	36·1	77·2	22·8	22·4	20·7–24·7
T2	1506	47·0	53·0	15·8	14·8–17·0	7·1	6·3–7·9	23·9	76·1	52·6	47·4	68·0	32·0	22·0	20·5–24·1
T3	1441	48·4	51·6	15·7	14·9–16·9	7·1	6·3–8·0	22·2	77·8	38·9	61·1	58·3	41·7	21·8	20·4–23·6
*P*-value		0·016		0·792		0·687		0·017		<0·001		<0·001		<0·001	
Children’s Dietary Inflammatory Score (C-DIS)															
T1	1573	49·7	50·3	15·8	14·8–17·1	7·4	6·7–8·3	27·0	73·1	57·9	42·1	71·5	28·5	22·3	20·5–24·5
T2	1572	49·9	50·1	15·8	14·9–17·0	7·1	6·3–7·0	21·4	79·0	52·8	47·2	69·2	30·0	22·2	20·6–24·4
T3	1572	48·2	51·8	15·7	14·8–16·8	6·8	6·0–7·6	24·7	75·3	47·0	53·0	64·7	35·3	21·7	20·3–23·6
*P*-value		0·592		0·043		<0·001		0·001		<0·001		<0·001		<0·001	
Children’s Eatwell Guide (C-EWG) score															
T1	1774	52·9	47·1	15·9	14·9–17·1	7·4	6·7–8·2	24·0	76·0	56·6	43·4	73·5	26·5	22·2	20·5–24·4
T2	2304	47·2	52·8	15·7	14·8–17·0	6·9	6·2–7·9	24·1	76·0	51·6	48·4	66·7	33·4	22·0	20·5–24·1
T3	639	46·8	53·2	15·6	14·8–16·7	6·9	6·0–7·8	26·4	73·6	44·9	55·1	60·8	39·2	21·8	20·4–23·8
*P*-value		0·001		0·013		<0·001		0·428		<0·001		<0·001		0·003	
Obesogenic-dietary pattern (DP)															
T1	1572	46·8	53·2	15·8	14·9–17·0	7·0	6·2–7·8	22·8	77·2	42·2	57·8	62·4	37·6	22·3	20·7–24·5
T2	1572	48·2	51·8	15·8	14·8–16·9	7·0	6·2–7·8	24·3	75·7	54·3	45·7	69·5	30·5	22·0	20·5–24·2
T3	1573	52·8	47·2	15·7	14·8–17·1	7·4	6·6–8·3	26·0	74·0	61·2	38·8	73·3	26·7	21·9	20·3–23·8
*P*		0·002		0·374		<0·001		0·126		<0·001		<0·001		0·001	

ALSPAC, Avon Longitudinal Study of Parents and Children; IQR, interquartile range (25th percentile and 75th percentile); CSE, Certificate of Secondary Education; T, tertile.

All percentage values are calculated as row percentages.

The participants excluded from the study due to missing exposure and outcome data were more likely to be male, to have lower socio-economic status and have mothers with lower educational attainment, compared with those children with complete data who were included in the final analysis (online Supplementary Table 4). Participants who were excluded generally had a similar mean PWV and cIMT at 17 and 24 years as those who were included in the analyses.

The mean (sd) cIMT (mm) was 0·47 (0·04) at 17 years and 0·46 (0·05) at 24 years, and the mean (sd) PWV (m/s) was 5·76 (0·70) at 17 years and 6·31 (1·09) at 24 years. The mean values of cIMT and PWV at 17 and 24 years according to tertiles of the DQ *Z*-score are shown in Table 3. In general, there were no differences in the mean cIMT and PWV between the tertiles, apart from for PWV at 17 years which was marginally higher in the bottom tertile of the Obesogenic-DP compared with the top tertile.

**Table 3.** Mean carotid intima-media thickness and pulse wave velocity in the ALSPAC participants at 17 and 24 years, according to tertile of the diet quality *Z*-scores

Tertiles of dietary pattern scores at 7 years	Cardiofunctional markers at 17 years						Cardiofunctional markers at 24 years					
	*n*	cIMT, mm mean	sd	*n*	PWV m/s mean	sd	*n*	cIMT, mm mean	sd	*n*	PWV m/s mean	sd
All participants	3203	0·47	0·04	2782	5·76	0·70	1382	0·46	0·05	1636	6·31	1·09
Children’s relative Mediterranean-style diet (C-rMED) score												
T1	1451	0·47	0·04	1262	5·78	0·72	606	0·46	0·05	712	6·31	1·02
T2	984	0·48	0·05	848	5·78	0·73	427	0·46	0·05	506	6·39	1·19
T3	768	0·48	0·04	672	5·71	0·62	349	0·46	0·05	418	6·24	1·08
*P*-value		0·808			0·289			0·886			0·338	
Dietary Approaches to Stop Hypertension (DASH) diet score												
T1	1127	0·47	0·04	979	5·79	0·71	460	0·46	0·05	541	6·35	1·06
T2	1023	0·47	0·05	894	5·75	0·71	453	0·46	0·05	547	6·26	1·05
T3	1053	0·48	0·05	909	5·75	0·69	469	0·46	0·05	548	6·33	1·15
*P*-value		0·362			0·208			0·090			0·305	
Children’s Dietary Inflammatory Score (C-DIS)												
T1	1040	0·47	0·04	903	5·79	0·71	445	0·46	0·05	516	6·33	1·03
T2	1054	0·48	0·05	932	5·77	0·71	456	0·46	0·05	545	6·32	1·15
T3	1109	0·48	0·05	947	5·74	0·69	481	0·46	0·05	575	6·29	1·09
*P*-value		0·683			0·227			0.689			0·707	
Children’s Eatwell Guide (C-EWG) score												
T1	1204	0·48	0·04	1058	5·79	0·72	486	0·46	0·05	582	6·32	1·09
T2	1539	0·48	0·04	1326	5·75	0·71	672	0·46	0·05	806	6·33	1·09
T3	460	0·47	0·04	398	5·73	0·63	224	0·45	0·05	248	6·24	1·12
*P*-value		0·170			0·276			0·141			0·280	
Obesogenic-dietary pattern (DP)												
T1	1158	0·47	0·05	1001	5·72	0·65	488	0·46	0·05	585	6·27	1·12
T2	1044	0·48	0·04	918	5·77	0·72	467	0·46	0·05	553	6·31	1·08
T3	1001	0·47	0·04	863	5·81	0·73	427	0·46	0·05	498	6·37	1·106
*P*		0·745			0·057			0·945			0·224	

ALSPAC, Avon Longitudinal Study of Parents and Children; cIMT, carotid intima-media thickness; PWV, pulse wave velocity; T, tertiles.

The results from the fully adjusted multivariable regression models assessing the association between the five DQ *Z*-scores at 7, 10 and 13 years of age and cIMT and PWV at 17 and 24 years are presented in Tables 4–6. None of the DQ scores at 7, 10 or 13 years were associated with cIMT at 17 or 24 years. A higher C-rMED score at 7 years was associated with a lower PWV at 17 years (*β* −0·07; 95 % CI (−0·14, −0·01) for high *v*. low C-rMED score). A higher Obesogenic-DP score at 7 and 10 years was associated with a higher PWV at 17 years (*β* 0·07 (95 % (0·01, 0·13)) and *β* 0·10 (95 % CI (0·04, 0·16)) for high *v*. low Obesogenic-DP score, respectively). The C-DIS score was associated with a lower PWV at 17 years: *β* − 0·06 (95 % CI (−0·12, −0·01)) for high *v*. low C-DIS score. The other DP scores were not associated with PWV at 17 years, and none of the DP scores at 7, 10 or 13 years were associated with PWV at 24 years. Overall, a similar pattern of results was observed in the analyses between the DQ total *Z*-scores and PWV and cIMT at 17 and 24 years (online Supplementary Table 5), whereby the Obesogenic-DP total *Z*-score and C-DIS total *Z*-score were associated with PWV at 17 years. In contrast, the C-rMED total *Z*-score was not associated with PWV at 17 years.

**Table 4.** Multivariable linear regression models for the relationship between the DQ scores at 7 years and carotid intima-media thickness and pulse wave velocity at 17 and 24 years, using imputed datasets in the ALSPAC cohort

Tertiles of dietary pattern	Carotid intima-media thickness (cIMT), mm								Pulse wave velocity (PWV), m/s							
	@17 years				@24 years				@17 years				@24 years			
	*n*	*β*	95 % CI*	*P*_for trend_	*n*	*β*	95 % CI*	*P*_for trend_	*n*	*β*	95 % CI*	*P*_for trend_	*n*	*β*	95 % CI*	*P*_for trend_
C-rMED *Z*-score at 7 years																
Low	1451	Reference			606	Reference			1262	Reference			712	Reference		
Medium	984	0·00	−0·00, 0·00		427	0·00	−0·00, 0·00		848	−0·00	−0·06,0·05		506	0·09	−0·02, 0·21	
High	768	0·00	−0·00, 0·00	0·685	349	0·00	−0·00, 0·01	0·441	672	−0·07	−0·14, −0·01	0·033	418	−0·09	−0·22, 0·04	0·339
Per 1-unit increment†	3203	0·00	−0·00, 0·00	0·305	1382	0·00	−0·00 0·00	0·258	2782	−0·03	−0·05, −0·00	0·030	1636	−0·02	−0·07, 0·03	0·433
DASH *Z*-score at 7 years																
Low	1127	Reference			460	Reference			979	Reference			541	Reference		
Medium	1023	0·00	−0·00, 0·01		453	0·00	−0·00, 0·01		894	−0·02	−0·08, 0·04		547	−0·08	−0·21, 0·05	
High	1053	0·00	−0·00, 0·01	0·083	469	0·01	−0·00, 0·01	0·094	909	−0·03	−0·09, 0·03	0·292	548	−0·04	−0·17, 0·09	0·512
Per 1-unit increment†	3203	0·02	0·00, 0·00	0·021	1382	0·00	−0·00, 0·00	0·148	2782	−0·01	−0·04, 0·01	0·380	1636	−0·02	−0·07, 0·04	0·513
C-EWG *Z*-score at 7 years																
Low	1204	Reference			486	Reference			1058	Reference			582	Reference		
Medium	1539	0·00	−0·00, 0·00		672	0·00	−0·00, 0·01		1326	−0·02	−0·07, 0·04		806	0·04	−0·07, 0·15	
High	460	−0·00	−0·00, 0·00	0·332	224	−0·00	−0·01, 0·00	0·580	398	−0·05	−0·13, 0·03	0·210	248	−0·05	−0·21, 0·11	0·776
Per 1-unit increment†	3203	−0·00	−0·00, 0·00	0·318	1382	−0·00	−0·0, 0·00	0·680	2782	−0·02	−0·05, 0·00	0·052	1636	−0·02	−0·07, 0·03	0·390
C-DIS *Z*-score at 7 years																
Low	1040	Reference			445	Reference			903	Reference			516	Reference		
Medium	1054	0·00	−0·00, 0·01		456	0·00	−0·00, 0·01		932	−0·02	−0·08, 0·04		545	−0·05	−0·17, 0·08	
High	1109	0·00	−0·00, 0·01	0·538	481	0·00	−0·01, 0·01	0·794	947	−0·05	−0·11, 0·01	0·104	575	−0·09	−0·21, 0·04	0·195
Per 1-unit increment†	3203	0·00	−0·00, 0·00	0·377	1382	0·00	−0·00, 0·00	0·654	2782	−0·13	−0·03, 0·00	0·133	1636	−0·03	−0·06, 0·01	0·133
Obesogenic-DP at 7 years																
Low	1158	Reference			488	Reference			1011	Reference			585	Reference		
Medium	1044	0·00	−0·00, 0·01		467	−0·00	−0·01, 0·01		918	0·05	−0·01, 0·11		553	0·07	−0·05, 0·19	
High	1011	−0·00	−0·00, 0·00	0·646	427	−0·00	−0·01, 0·01	0·803	863	0·07	0·01, 0·13	0·028	498	0·11	−0·02, 0·24	0·084
Per 1-unit increment†	3203	−0·00	−0·00, 0·00	0·446	1382	−0·00	−0·00, 0·00	0·677	2782	0·03	0·01, 0·06	0·004	1636	0·05	−0·00, 0·10	0·061

DQ, diet quality; ALSPAC, Avon Longitudinal Study of Parents and Children; C-rMED, children’s relative Mediterranean-style diet; DASH, Dietary Approaches to Stop Hypertension; C-EWG: children’s Eatwell Guide; C-DIS, children’s Dietary Inflammatory Score; Obesogenic-DP, Obesogenic-dietary pattern (derived from reduced rank regression methods).

* *β* coefficients derived from multivariable regression model adjusted for sex, dietary misreporting, maternal highest education level, family highest social class, puberty timing and physical activity level at 11 years (for analysis of dietary patterns at 7 and 10 years) and physical activity at 13 years (for analysis of dietary patterns at 13 years).

† Estimated mean change in cIMT or PWV associated with a 1-point increase in dietary pattern *Z*-score.

**Table 5.** Multivariable linear regression models for the relationship between the DQ scores at 10 years and carotid intima-media thickness and pulse wave velocity at 17 and 24 years, using imputed datasets in the ALSPAC cohort

Tertiles of dietary pattern	Carotid intima-media thickness (cIMT), mm								Pulse wave velocity (PWV), m/s							
	@17 years				@24 years				@17 years				@24 years			
	*n*	*β*	95 % CI*	*P*_for trend_	*n*	*β*	95 % CI*	*P*_for trend_	*n*	*β*	95 % CI*	*P*_for trend_	*n*	*β*	95 % CI*	*P*_for trend_
C-rMED *Z*-score at 10 years																
Low	1534	Reference			632	Reference			1316	Reference			712	Reference		
Medium	923	−0·00	−0·00, 0·00		400	0·01	−0·00, 0·01		813	0·01	−0·05, 0·07		506	−0·03	−0·16, 0·09	
High	746	0·00	−0·00, 0·00	0·926	350	0·00	−0·00, 0·01	0·348	653	0·02	−0·04, 0·09	0·477	418	0·00	−0·12, 0·13	0·987
Per 1-unit increment†	3203	0·00	−0·00, 0·00	0·352	1382	0·00	−0·00, 0·00	0·379	2782	−0·00	−0·03,0·02	0·865	1636	0·01	−0·04, 0·06	0·733
DASH *Z*-score at 10 years																
Low	1144	Reference			464	Reference			1005	Reference			546	Reference		
Medium	1053	−0·00	−0·00, 0·00		468	0·00	−0·00, 0·01		898	0·07	0·01, 0·13		559	−0·02	−0·15, 0·10	
High	1006	0·00	−0·00, 0·01	0·361	450	0·00	−0·00, 0·01	0·599	879	−0·02	−0·08,0·04	0·581	531	−0·06	−0·19, 0·07	0·361
Per 1-unit increment†	3203	0·00	−0·00, 0·00	0·098	1382	0·00	−0·00, 0·00	0·691	2782	0·01	−0·04, 0·01	0·297	1636	−0·00	−0·06, 0·05	0·921
C-EWG *Z*-score at 10 years																
Low	1301	Reference			531	Reference			1133	Reference			627	Reference		
Medium	901	0·00	−0·00, 0·01		395	0·00	−0·01, 0·01		788	0·01	−0·05, 0·07		477	−0·07	−0·20, 0·06	
High	1001	0·00	−0·00, −0·00	0·712	456	0·00	−0·00, 0·01	0·500	861	−0·03	−0·09, 0·03	0·372	532	−0·07	−0·20, 0·06	0·279
Per 1-unit increment†	3203	0·00	−0·00, 0·00	0·923	1382	0·00	−0·00, 0·00	0·417	2782	−0·00	−0·03, 0·02	0·724	1636	−0·01	−0·06, 0·04	0·624
C-DIS *Z*-score at 10 years																
Low	1029	Reference			445	Reference			895	Reference			528	Reference		
Medium	1069	−0·00	−0·00, 0·00		459	0·00	−0·01, 0·01		927	−0·05	−0·11, 0·01		539	0·12	−0·01, 0·24	
High	1105	−0·00	−0·01, 0·00	0·356	478	0·00	−0·01, 0·01	0·927	960	−0·06	−0·12,−0·01	0·017	569	0·01	−0·12, 0·13	0·965
Per 1-unit increment†	3203	−0·00	−0·00, 0·00	0·802	1382	0·00	−0·00, 0·00	0·845	2782	−0·02	−0·04, −0·00	0·017	1636	−0·00	−0·04, 0·03	0·932
Obesogenic-DP at 10 years																
Low	1121	Reference			509	Reference			972	Reference			601	Reference		
Medium	1056	0·00	−0·00, 0·00		426	−0·01	−0·01, 0·00		917	0·08	0·03, 0·14		522	0·01	−0·11, 0·14	
High	1026	−0·00	−0·00, 0·00	0·746	447	−0·01	−0·01, 0·00	0·047	893	0·10	0·04, 0·16	0·001	513	0·01	−0·12, 0·14	0·853
Per 1-unit increment†	3203	−0·00	−0·00, 0·00	0·867	1382	−0·00	−0·00, 0·00	0·068	2782	0·03	0·01, 0·05	0·003	1636	−0·01	−0·05, 0·03	0·679

DQ, diet quality; ALSPAC, Avon Longitudinal Study of Parents and Children; C-rMED, children’s Relative Mediterranean-style diet; DASH: Dietary Approaches to Stop Hypertension; C-EWG: children’s Eatwell Guide; C-DIS, children’s Dietary Inflammatory Score; Obesogenic-DP, Obesogenic-dietary pattern (derived from reduced rank regression methods).

* *β* coefficients derived from multivariable regression model adjusted for sex, dietary misreporting, maternal highest education level, family highest social class, puberty timing and physical activity level at 11 years (for analysis of dietary patterns at 7 and 10 years) and physical activity at 13 years (for analysis of dietary patterns at 13 years).

† Estimated mean change in cIMT or PWV associated with a 1-point increase in dietary pattern *Z*-score.

**Table 6.** Multivariable linear regression models for the relationship between the DQ scores at 13 years and carotid intima-media thickness and pulse wave velocity at 17 and 24 years, using imputed datasets in the ALSPAC cohort

Tertiles of dietary pattern	Carotid intima-media thickness (cIMT), mm								Pulse wave velocity (PWV), m/s							
	@17 years				@24 years				@17 years				@24 years			
	*n*	*β*	95 % CI*	*P*_for trend_	*n*	*β*	95 % CI*	*P*_for trend_	*n*	*β*	95 % CI*	*P*_for trend_	*n*	*β*	95 % CI*	*P*_for trend_
C-rMED *Z*-score at 13 years																
Low	1075	Reference			434	Reference			943	Reference			520	Reference		
Medium	986	0·00	−0·00, 0·01		443	0·00	−0·00, 0·01		848	0·00	−0·06, 0·07		519	−0·02	−0·15, 0·11	
High	1142	0·00	−0·00, 0·01	0·278	505	0·00	−0·00, 0·01	0·453	991	0·00	−0·6, 0·06	0·927	597	−0·07	−0·20, 0·06	0·289
Per 1-unit increment†	3203	0·00	−0·00, 0·00	0·191	1382	0·00	−0·00, 0·00	0·318	2782	0·01	−0·03, 0·01	0·583	1636	−0·04	−0·09, 0·02	0·183
DASH *Z*-score at 13 years																
Low	1177	Reference			491	Reference			1037	Reference			579	Reference		
Medium	1037	0·00	−0·00, 0·01		435	0·00	−0·00, 0·01		893	0·02	−0·04, 0·08		524	0·08	−0·05, 0·21	
High	989	0·00	−0·00, 0·01	0·312	456	0·00	−0·00, 0·01	0·430	852	0·01	−0·05, 0·07	0·771	533	−0·02	−0·15, 0·11	0·806
Per 1-unit increment†	3203	0·00	−0·00, 0·00	0·260	1382	0·00	−0·00, 0·00	0·164	2782	−0·00	−0·03, 0·02	0·842	1636	−0·03	−0·09, 0·02	0·215
C-EWG *Z*-score at 13 years																
Low	1833	Reference			790	Reference			1610	Reference			939	Reference		
Medium	727	−0·00	−0·01, 0·00		314	−0·00	−0·01, 0·01		634	0·02	−0·04, 0·08		373	−0·07	−0·20, 0·06	
High	643	−0·00	−0·01, 0·00	0·133	278	−0·00	−0·01, 0·00	0·582	538	−0·05	−0·12, 0·01	0·209	324	−0·07	−0·21, 0·07	0·254
Per 1-unit increment†	3203	0·00	−0·00, 0·00	0·310	1382	0·00	−0·00, 0·00	0·961	2782	−0·00	−0·03, 0·02	0·720	1636	−0·03	−0·08, 0·03	0·312
C-DIS *Z*-score at 13 years																
Low	1020	Reference			438	Reference			895	Reference			518	Reference		
Medium	1099	0·00	−0·00, 0·01		486	0·00	−0·01, 0·01		952	0·03	−0·03, 0·09		572	0·05	−0·08, 0·18	
High	1084	−0·00	−0·00, 0·00	0·747	458	0·00	−0·01, 0·01	0·934	935	0·01	−0·05, 0·07	0·737	546	−0·06	−0·19, 0·08	0·338
Per 1-unit increment†	3203	−0·00	−0·00, 0·00	0·892	1382	0·00	−0·00, 0·00	0·841	2782	−0·00	−0·02, 0·01	0·587	1636	−0·01	−0·05, 0·02	0·481
Obesogenic-DP at 13 years																
Low	1128	Reference			491	Reference			972	Reference			572	Reference		
Medium	1076	0·00	−0·00, 0·00		452	−0·01	−0·01, −0·00		928	−0·03	−0·09, 0·03		547	0·00	−0·12, 0·13	
High	999	−0·00	−0·01, 0·00	0·474	439	−0·00	−0·01, 0·00	0·343	882	0·03	−0·03, 0·10	0·336	517	0·04	−0·09, 0·17	0·537
Per 1-unit increment†	3203	−0·00	−0·00, 0·00	0·690	1382	−0·00	−0·00, 0·00	0·441	2782	0·01	−0·01, 0·02	0·481	1636	−0·01	−0·05, 0·03	0·552

DQ, diet quality; ALSPAC, Avon Longitudinal Study of Parents and Children; C-rMED, children’s Relative Mediterranean-style diet; DASH, Dietary Approaches to Stop Hypertension; C-EWG, children’s Eatwell Guide score; C-DIS, children’s Dietary Inflammatory Score; Obesogenic-DP, Obesogenic-dietary pattern (derived from reduced rank regression methods).

* *β* coefficients derived from multivariable regression model adjusted for sex, dietary misreporting, maternal highest education level, family highest social class, puberty timing and physical activity level at 11 years (for analysis of dietary patterns at 7 and 10 years) and physical activity at 13 years (for analysis of dietary patterns at 13 years).

† Estimated mean change in cIMT or PWV associated with a 1-point increase in dietary pattern *Z*-score.

In sensitivity analyses, additionally adjusting for smoking intensity and alcohol consumption at 17 years in the models where there was an association (between the Obesogenic-DP at 7 and 10 years and PWV at 17 years, the C-rMED score at 7 years and PWV at 17 years and the c-DIS score at 10 years and PWV at 17 years), there was no change to the effect estimates or CI for the relationships (data not tabulated). When the main regression models were repeated in complete case analyses, similar results were observed, with the exception of the association between the C-rMED score at 7 years and PWV at 17 years, which was weaker (online Supplementary Tables 6 to 11). *Post hoc* analyses exploring which of the individual food groups within the C-rMED at 7 years were associated with PWV at 17 years showed that fruit and cereal intakes were related to a decrease in PWV (*β* −0·10 (95 % CI (−0·17, −0·04)) for high *v*. low tertile of fruit intake, and *β* −0·07 (95 % CI (−0·13, −0·01)) for high *v*. low tertile of cereal intake). Although there was no evidence of an association for the remaining food groups within the C-rMED, the *β* coefficients were in the postulated/favourable direction (data not tabulated).

## Discussion

To the best of our knowledge, this is the first prospective study to investigate and compare the associations between several established DQ indices throughout childhood and arterial stiffness and cIMT in adolescence and early adulthood. The main finding was that an Obesogenic-DP, characterised by high energy density, high fat and free sugars and low fibre, during childhood (7–10 years) was related to increased arterial stiffness in adolescence. Consistent with this, Mediterranean-style and anti-inflammatory DP at 7 or 10 years were related to decreased arterial stiffness. In this cohort, none of the DQ scores measured in childhood were related to cIMT at 17 or 24 years.

### Obesogenic-dietary pattern and pulse wave velocity

Our findings related to the energy-dense, high-fat, high free sugars and low-fibre DP showed that each 1-unit increment in this Obesogenic-DP was related to a 0·03 m/s increase in PWV at 17 years. Few studies have explored whether childhood DP identified using RRR are associated with arterial stiffness. However, a prospective study of ∼230 youths with type 1 diabetes in the USA identified a DP using RRR, characterised by high intakes of sugar-sweetened beverages, diet soda, eggs, potatoes and high-fat meats, was positively associated with arterial stiffness measured via augmentation index^(26)^. This DP was also related to PWV, but the association was lost once confounders were taken into account. Epidemiological studies using other data-driven methods to identify DP have also provided mixed results. A cross-sectional study of 389 9–11-year-old children from New Zealand identified a ‘snack’ pattern and a ‘fruit and vegetable’ pattern using principal component analysis, but neither were associated with arterial stiffness measured using PWV and augmentation index^(54)^. In contrast, the Young Finns Study found that long-term adherence (from 3–18 years) to a traditional DP (characterised by high consumption of rye, potatoes, butter, sausages, milk and coffee) using PCA increased cIMT in adult males (aged about 30 years), but not females^(22)^. The Northern Ireland Young Hearts study found that 20–25-year-old participants adhering most closely to the ‘healthy’ DP identified using PCA had a lower PWV, although this association was not observed in their longitudinal analyses^(55)^. A prospective cohort study of 1026 adults also found that a high meat and alcohol, low-fibre and micronutrient-poor DP derived using PCA was related to increased stiffening of the large arteries^(11)^. However, it is difficult to directly compare results between studies analysing *a posteriori* DP, as different styles of DP are often identified due to cultural differences in dietary habits between populations and in the case of RRR due to differences in the response variables available for selection.

### Children’s relative Mediterranean-style diet score and pulse wave velocity

Our finding that a more Mediterranean-style diet at 7 years was related to lower PWV at 17 years is in line with the Amsterdam Growth and Health Longitudinal Study which reported a favourable association between a Mediterranean diet in adolescence and adulthood (24 years follow-up) and arterial stiffness in adulthood^(18)^. Furthermore, a cross-sectional study of 227 12-year-old children living in Greece found that a Mediterranean diet correlated with the augmentation index, independent of obesity^(56)^. In contrast, a longitudinal study in youths with type 1 diabetes (*n* 486) in the USA did not find any association between a Mediterranean diet and arterial stiffness measured with PWV or augmentation index^(25)^. However, DQ was generally poor to moderate in this population, so there may not have been close enough adherence to a Mediterranean-style diet to impact vascular markers. A key attribute of the Mediterranean DP is an abundance of fruit and vegetables, and the Young Finns Study has shown that a regular high intake of fruit and vegetables from childhood to adulthood was related to better arterial elasticity in adulthood^(17)^.

### Children’s Dietary Inflammatory Score and pulse wave velocity

Our study also showed that a more anti-inflammatory diet (compared with a pro-inflammatory diet) at about 10 years of age was related to lower arterial stiffness 7 years later. To the best of our knowledge, this is the first cohort study to assess this association in this age group. However, our results are supported by previous studies in adults which also found favourable associations between the inflammatory potential of the diet and vascular function. For instance, a cohort of ninety overweight and sedentary adults from Columbia demonstrated that a more anti-inflammatory dietary score was inversely correlated with PWV (*r* = −0·437, *P* < 0·05)^(57)^. In cross-sectional study of 2644 middle-aged and elderly women in China, a more pro-inflammatory diet was related to higher brachial ankle PWV in participants with diabetes and prediabetes, but not in females with normal glucose homoeostasis^(58)^.

### Children’s Dietary Approaches to Stop Hypertension score and pulse wave velocity

In this cohort study, we did not observe any association between the C-DASH diet and arterial stiffness at any age. Previous epidemiological research in youth and adults has reported mixed results regarding this relationship. A cross-sectional study of fifty-six healthy young adults in the USA found no link between two types of DASH diet scores and PWV or augmentation index^(59)^. Similarly, a cross-sectional study of 10–30-year-olds with type 1 diabetes reported no association between three DQ indices (the DASH diet, Alternative Healthy Eating Index and Mediterranean diet) and PWV^(25)^. In contrast, a British cohort study (*n* 1409) demonstrated that greater adherence to the DASH diet over the life course (from 36–65 years) was related to both cIMT and PWV^(60)^. Differences in study design, participant age groups and health status and construction of the DASH diet may be possible explanations for differences in findings between studies. The fact we observed an association between the C-rMED and PWV at 17 years but not the C-DASH score could be because the C-rMED additionally captures differences in fish intake and lipid profiles, which may be relevant for maintaining arterial elasticity.

### Children’s Eatwell Guid score and pulse wave velocity

There was also no evidence that closer adherence to UK dietary guidelines during childhood was related to a decrease in PWV in adolescence/early adulthood in our study. An explanation could be the construction of the C-EWG score which dichotomises children’s intake of each food and nutrient within the score into either above or below the recommended amount, with no additional level of sensitivity to distinguish children’s intakes that were close to or far from the dietary recommendation. This is relevant because for many foods and nutrients a large proportion of children did not meet the dietary recommendations, although their intakes varied considerably above or below the thresholds. To our knowledge, this is the first study to evaluate the relationship between adherence to UK dietary guidelines and vascular damage in children and adolescents, so direct comparisons with other studies are not possible. However, previous research has assessed adherence Dietary Guidelines for Americans and arterial stiffness in adults, producing mixed results. Although we did not find an association between the C-EWG and PWV, previous research from our group showed that meeting more UK dietary recommendations at 7 and 10 years had favourable effects on body fat, insulin resistance and mean arterial blood pressure, and an overall cardiometabolic risk score at 24 years^(30)^.

### Diet quality score and pulse wave velocity and carotid intima-media thickness

The associations we observed between the DQ *Z*-scores (C-rMED, C-DIS and Obesogenic-DP) and PWV at 17 years were not apparent when PWV was measured at 24 years, although the effect estimates were of similar magnitude and direction. Due to loss to follow-up, the number of participants with PWV data at 24 years was ∼40 % smaller than the sample size at 17 years (despite multiple imputation of missing covariate data), which may have reduced the study’s power to detect associations at 24 years. Furthermore, we did not observe any association between the DQ scores at 13 years and PWV at either age. This could be a result of more changeable (and more difficult to accurately capture) dietary habits at 13 years as children gain increased autonomy in food choices outside the home, partly due to the move from primary to secondary school. The 13-year-olds also had higher levels of misreporting of dietary intake (mainly under-reporting), although dietary misreporting was adjusted for in multivariable regression models.

In addition, none of the DQ *Z*-scores measured between 7 and 10 years were related to cIMT at 17 or 24 years. Previous studies exploring this relationship within these age groups is scare and has mainly focused on children at higher risk of CVD. For instance, a 1-year Mediterranean-style dietary intervention was carried out in thirty-six pre-pubertal hypercholesterolaemic children and reported a significant decrease in cIMT^(23)^. A cross-sectional study of 232 children/adolescents with congenital heart disease found that low-fat dairy DP derived from principal component analysis was inversely associated with cIMT (*β*: −0·024; 95 % CI (−0·04, −0·01))^(24)^. It is possible that in apparently healthy participants, as in our study, measuring cIMT at 17 and 24 years was still too early to detect significant changes in this outcome due to lack of variability in the sample given their age, or the sample size limited our power to detect an association. Indeed, a systematic review of observational studies in adults exploring the influence of *a posteriori* and *a prior*i DP (including the AHEI and DII) on cIMT concluded that in general a higher consumption of ‘healthy’ foods and lower consumption of ‘unhealthy’ foods were related to a decreased cIMT^(13)^. However, the majority of the studies were cross-sectional and the results from *a posteriori* derived DP were fairly heterogeneous^(13)^. In addition, two prospective cohort studies, each including over a thousand adults, reported that RRR derived healthy and unhealthy DP in their cohorts were not associated with cIMT^(11,15)^. Therefore, larger and longer-term prospective studies are still needed to better understand the role of DQ during childhood on future cIMT and PWV.

### Biological mechanisms

There are multiple potential mechanisms that could explain how a Mediterranean-style and anti-inflammatory diets could preserve arterial elasticity and how an energy-dense, high-fat, high free sugars, low-fibre DP could increase arterial stiffening. One of the distinguishing features of the Mediterranean diet (and which differentiates it from the DASH diet) is regular consumption of fish and seafood, which are rich in long-chain *n*-3 PUFA. The benefits of *n*-3 PUFA on arterial stiffness are not entirely clear but may be linked to the incorporation of *n*-3 into cells that provokes *n*-6-derived pro-inflammatory eicosanoids which lower platelet aggregation, inflammation and vasoconstriction^(10)^. Other components of the Mediterranean diet, such as fruit and vegetables, olive oil, and wholegrain cereals and pulses, are rich in antioxidants and phytochemicals such as polyphenols, which exhibit a diverse range of cardiovascular health benefits. In terms of arterial health, polyphenols can increase the bioavailability of nitric oxide, which influences large artery dispensability and therefore may be effective in reducing arterial stiffness^(10)^.

Anti-inflammatory diets can protect against chronic low-grade inflammation by increasing the production of anti-inflammatory cytokines while reducing pro-inflammatory cytokines, which in turn can lower oxidative stress (reduce production of reactive oxygen species) and increase cellular antioxidant capacity^(61)^. These processes may protect against the age-related decline in arterial elasticity caused by structural changes in the arterial walls from fibrosis, fragmentation and degradation of elastin^(57)^. The Obesogenic-DP had high factor loadings of refined carbohydrates and sugary foods and drinks which could increase pro-inflammatory markers and produce a state of chronic low-grade inflammation^(62)^, leading to detrimental effects on vascular function.

### Limitations and strengths

Our study has several limitations. The first of these is loss to follow-up, which is a common issue in long-term prospective studies, particularly with repeated measures of clinical data. This resulted in attrition bias whereby children with a lower socio-economic status were under-represented in the final study sample. Furthermore, previous research in ALSPAC has shown that children’s DQ is correlated with socio-economic factors^(63)^, meaning that children with more unhealthy DP were also likely to be under-represented in our analysis. Although this limits the generalisability of our results to the general population, it would not affect the internal validity of the associations we observed. A further limitation is the use of self-reported 3-d diet diaries to assess dietary intake, which could introduce reporting error and recall bias. However, we included plausibility of dietary reporting as a potential confounder in all multivariable analyses, and diet diaries have been shown to be less prone to misreporting than FFQ^(64)^. The study outcomes (PWV and cIMT) could also be affected by measurement error; however, this would be expected to be non-differential and would therefore only bias results towards to the null. Although we took into account a wide range of potential confounders, we cannot disregard residual confounding due to measurement error in these covariates or other unknown confounding factors not included. Additionally, as this is an observational study, we cannot assume causality in the observed associations. Finally, none of the DQ scores included the global intake of ultra-processed food, which is an emerging risk factor for developing CVD^(65)^ and therefore could be linked to cIMT. Further research could investigate a DP capturing intake of ultra-processed food and cIMT and PWV.

Our study also has several major strengths, including the prospective nature spanning up to 17 years of follow-up, along with the relatively large sample size for a birth cohort study with PWV and cIMT cardio endpoints. These outcomes are useful pre-clinical measures of CVD risk in young adults because they are strong predictors of future CVD^(4,7,8)^. We were also able to evaluate DQ at three different time points throughout childhood, which is relevant as assessing the age that dietary factors can begin to influence the development of atherosclerosis can be useful for the timing of CVD preventative strategies and give insights into the aetiology of CVD. In addition, by exploring several different measures of DQ, we were able to explore which particular DP were associated with arterial stiffness and thickness. Furthermore, analysing DP can be advantageous compared with single foods/nutrients as it can account for synergistic relationships between dietary components and capture the accumulative effects of groups of foods/nutrients whose individual health effect may be undetectable. This was illustrated in our *post hoc* analyses exploring associations between individual components of the C-rMED and PWV.

A further strength is that we imputed missing confounder data which helped minimise attrition bias due to missing follow-up data and improve efficiency and precision of association estimates^(66)^. Nevertheless, future studies assessing these associations using larger sample sizes could provide more precise effect estimates.

### Conclusion

This cohort study found that an Obesogenic-DP at 7 and 10 years was related to greater arterial stiffness in adolescence, while a Mediterranean-style diet and an anti-inflammatory diet (both predominantly plant-based diets, rich in fibre, mono- and polyunsaturated fats, antioxidants and anti-inflammatory foods/nutrients) at 7 or 10 years, respectively, were related to less arterial stiffness. These novel research findings help fill the gap in our understanding on the influence of DQ during childhood on vascular dysfunction. The results add to epidemiological evidence on the health benefits of adopting more Mediterranean-style and anti-inflammatory diets from childhood, due to their potential to protect against vascular damage. This may also explain part of the beneficial effects these DP have on CVD. In addition, our findings showed that an energy-dense DP, high in fat and free sugars and low in fibre – a common feature of many of children’s diets in the UK^(42)^ – could be already contributing to the progression of arterial stiffening in adolescents. Overall, our findings highlight the importance of establishing healthy dietary habits early in life to protect against arterial stiffness, which is a strong predictor of CVD later in life.

## Supporting information

Buckland et al. supplementary material Buckland et al. supplementary material

## Supplementary material

For supplementary material/s referred to in this article, please visit https://doi.org/10.1017/S0007114523002763
