# Supplementary material for: Associations of childhood diet quality scores with arterial stiffness and carotid artery intima-media thickness in adolescence/early adulthood: findings from the ALSPAC cohort
Source: Br J Nutr. 2024 Jan 5;131(4):720–35. doi: 10.1017/S0007114523002763 (PMC10803818; doi:10.1017/S0007114523002763)
Supplement: Buckland et al. supplementary material [file S0007114523002763sup001.docx]

**Supplementary Material:**

**Associations of childhood diet quality scores with arterial stiffness and carotid artery intima-media thickness in adolescence/early adulthood: findings from the ALSPAC cohort**

Genevieve Buckland^1^, Kate Northstone^2^, Pauline M Emmett^1^ and Caroline M Taylor^1^

**^1^Centre for Academic Child Health, Bristol Medical School, University of Bristol**

**^2^**Department of Population Health Sciences**, Bristol Medical School, University of Bristol**

**Supplementary Methods**

The methodology used to construct the five diet quality scores for each participant, based on the dietary data that was available at 7, 10 and 13 years old, is detailed below.

*Children’s relative Mediterranean diet (C-rMED) score*

The C-rMED score is based on the intake of eight food groups or components: fruit, vegetables, pulses, cereals and cereal products, fish/seafood, dairy products, meat and meat products and a lipid ratio (sum of monounsaturated fatty acids (MUFA) and polyunsaturated fatty acids (PUFA) divided by saturated fatty acids (SFA) g/day) (1). Intake of each component was calculated as a function of total energy intake (g/4184 kJ per day (g/1000 kcal per day)) (2). For fruit, vegetables, cereals, and dairy products a value of 0, 1, or 2 was assigned to the first, second, or third tertile of intake, respectively (higher intakes received higher scores). The scoring was reversed for meat intake so that higher intakes received lower scores. The lipid ratio was scored by assigning 0 and 1 points for intakes below and above the median. For legumes and fish/seafood a value of 0 was assigned to non-consumers (due to large proportions of non-consumers) and 1 and 2 points were assigned to intakes below and above the median for consumers, respectively. The points obtained from each of the components are summed together for each participant to produce a C-rMED score, with possible scores ranging from 0 to 15 (lowest to highest adherence). The C-rMED score was standardised by converting it to z-scores. This was repeated at each dietary data collection timepoint.

*Children’s Dietary Inflammatory score (C-DIS)*

The C-DIS was previously adapted from the C-DII which originated from the Dietary Inflammatory Index (DII) (3). It is a tool for estimating the overall inflammatory potential of the diet. The C-DIS includes 24 components: energy, carbohydrate, protein, total fat, saturated fat, monounsaturated fat, polyunsaturated fat, dietary cholesterol, fibre, vitamin A, vitamin B6, vitamin B12, vitamin C, vitamin D, vitamin E, folic acid, beta-carotene, thiamine, riboflavin, niacin, iron, magnesium, zinc and selenium. Each dietary component is divided by total energy, standardised using z-scores and then multiplied by its corresponding positive or negative inflammatory weight (previously calculated and published (3)) which reflect their pro-inflammatory or anti-inflammatory properties. The inflammatory z-score from each of the 24 dietary components are summed together for each participant to give their total C-DIS. The C-DIS z-score was multiplied by −1, to align the direction of values for increasing score representing a healthier diet (as in the other a priori scores).

*Dietary Approaches to Stop Hypertension (DASH) diet score*

A DASH diet score was constructed based on previously published methods (4). The DASH diet score incorporates the intake of eight foods/nutrients defined within a DASH-style dietary pattern: fruit, vegetables, nuts and legumes, wholegrains, low-fat dairy products (the healthy components) and red and processed meat, sodium and non-milk extrinsic sugars (NMES – similar to free sugars) (the unhealthy components). Each food/nutrient was assessed as a function of total energy intake (g/4184 kJ per day (g/1000 kcal per day)) (2). The intakes of each food/nutrient were divided into quintiles and each participant received a score of 1-5 depending on which quintile their intake fell into. For the healthy foods/nutrients, scores of 1-5 were assigned to quintiles 1-5 respectively (highest quintile of intake receiving highest points), while for the unhealthy foods/nutrients the scoring was reversed and scores of 5-1 were assigned to quintiles 1-5 (highest quintile of intake receiving lowest scores). The score obtained from each of the eight foods/nutrients were added together to create the DASH diet score for each child at each age, with a potential range of 8-40. The DASH diet score was then standardised by converting it to z-scores.

*Children’s Eatwell Guide Score (C-EWG score)*

The C-EWG score (5) includes nine foods/nutrients: total fat, saturated fat, free sugars, fibre, salt, fruit and vegetables, non-oily fish, oily fish and red/processed meat. The UK government dietary recommendations for these foods/nutrients are outlined in the Health Security Agency 2016 Report (6), and visually represented within the Eatwell Guide (7). Each participant’s intake of the nine foods/nutrients was dichotomised into adhering to (1 point) or not adhering to (0 points) the dietary guidelines, which when necessary were adapted for the different ages of the children (7-13 years) by proportionally scaling down adult to child portion sizes. The points obtained from each food/nutrient were summed for each participant to give the C-EWG score, which could range from 0 to 9 (none to all recommendations met). The full details of the construction of the C-EWG score have been published previously (8). The UK dietary recommendations (minimum intakes or constraints) for each of the C-EWG foods and nutrients at the corresponding ages of the participants are detailed in Supplementary Table 1. The C-EWG scores were standardised by converting them to z-scores.

*Obesogenic Dietary Pattern Score (Obesogenic-DP)*

The Obesogenic-DP used in this analysis was previously identified using reduced rank regression (RRR) in ALSPAC children at 7, 10 and 13 years of age by analysing which food groups within their diet explained the maximum variation in a set of four response variables hypothesised to be on the pathway between food consumption and obesity (9). The response variables included energy density (kJ of total food energy / total food weight (g)), percentage energy from fat, percentage energy from free sugars, and dietary fibre (non-starch polysaccharide) density (g/kJ). The RRR analysis considered 46 food groups as predictor variables within the model and produced factor loadings for each of these food groups, which quantified the extent to which they increased (positive values) or decreased (negative values) the energy density, free sugars, fat or fibre content of the diet. The final Obesogenic-DP z-score resulted in the combination of food groups that explained the maximum amount of shared variation in these response variables. A higher Obesogenic-DP z-score reflected a diet higher intakes of free sugars, fat and energy density and lower intakes of fibre (9).

**References**

1. Buckland G, Taylor CM, Emmett PM, Johnson L, Northstone K. Prospective association between a Mediterranean-style dietary score in childhood and cardiometabolic risk in young adults from the ALSPAC birth cohort. Eur J Nutr. 2022;61(2):737-52. doi:10.1007/s00394-021-02652-7

2. Willett W, Howe G, Kushi L. Adjustment for total energy intake in epidemiologic studie. Am J Clin Nutr. 1997;65. :S1220-S8.

3. Shivappa N, Steck SE, Hurley TG, Hussey JR, Hebert JR. Designing and developing a literature-derived, population-based dietary inflammatory index. Public Health Nutr. 2014;17(8):1689-96. doi:10.1017/S1368980013002115

4. Jones NRV, Forouhi NG, Khaw KT, Wareham NJ, Monsivais P. Accordance to the Dietary Approaches to Stop Hypertension diet pattern and cardiovascular disease in a British, population-based cohort. Eur J Epidemiol. 2018;33(2):235-44. doi:10.1007/s10654-017-0354-8

5. Buckland G, Northstone K, Emmett P, Taylor C. Adherence to UK dietary guidelines in school-aged children from the Avon Longitudinal Study of Parents and Children (ALSPAC) cohort. BJN. 2022;28:1-13.

6. Public Health England: Nutrition Science Team (2016) Government Dietary Recommendations: Government recommendations for energy and nutrients for males and females aged 1-18 years and 19+ years. <https://assets.publishing.service.gov.uk/government/uploads/system/uploads/attachment_data/file/618167/government_dietary_recommendations.pdf>. Accessed 23.09.21.

7. Public Health England (2018). The Eatwell Guide. <https://www.gov.uk/government/publications/the-eatwell-guide>. Accessed 23.09.21.

8. Buckland G, Taylor CM, Emmett PM, Northstone K. Prospective association between adherence to UK dietary guidelines in school-age children and cardiometabolic risk markers in adolescence/early adulthood in the Avon Longitudinal Study of Parents and Children (ALSPAC) cohort. British Journal of Nutrition. 2023:1-13. doi:10.1017/s0007114523000685

9. Ambrosini GL, Johns DJ, Northstone K, Emmett PM, Jebb SA. Free Sugars and Total Fat Are Important Characteristics of a Dietary Pattern Associated with Adiposity across Childhood and Adolescence. The Journal of nutrition. 2015;146(4):778-84. doi:10.3945/jn.115.224659

**Supplementary Table 1.** UK dietary recommendations for key nutrients and foods within the Eatwell Guide, including age-adjusted portion sizes calculated for the ALSPAC children at 7, 10 and 13 years old.

**References in footnotes of Table 1:**

1. Public Health England (2019) The Scientific Advisory Committee on Nutrition (SACN) Report on Saturated Fats and Health. https://www.gov.uk/government/publications/saturated-fats-and-health-sacn-report (accessed September 2021).

2. Public Health England (PHE) (2011) Scientific Advisory Committee on Nutrition: Dietary Reference Values for Energy. https://www.gov.uk/government/publications/sacn-dietaryreference-values-for-energy (accessed November 2021). Public Health England (PHE) (2015)

3.Carbohydrates and Health Report. The Scientific Advisory Committee on Nutrition Recommendations on Carbohydrates, Including Sugars and Fibre. https://www.gov.uk/government/publications/sacncarbohydrates-and-health-report (accessed September 2021).

4. Public Health England (PHE) (2003) Scientific Advisory Committee on Nutrition Advice on Salt and Recommended Dietary Reference Values (DRVs). https://www.gov.uk/ government/publications/sacn-salt-and-health-report (accessed September 2021).

5. Public Health England (2018) The Eatwell Guide. https://www. gov.uk/government/publications/the-eatwell-guide (accessed September 2021).

6. Jones LR, Steer CD, Rogers IS, et al. (2010) Influences on child fruit and vegetable intake: sociodemographic, parental and child factors in a longitudinal cohort study. Public Health Nutr 13, 1122–1130.

**Supplementary Table 2.** Comparison of characteristics of ALSPAC participants with imputed and observed data for pulse wave velocity (PWV) outcome analyses.

**Supplementary Table 3.** Comparison of characteristics of ALSPAC participants with imputed and observed data for carotid intima-media thickness (cIMT) outcome analyses.

**Supplementary Table 4.** Comparison of baseline characteristics and arterial functional variables at 17 years and 24 years in ALSPAC participants who had complete dietary data at all three ages (7, 10 and 13 years) and outcome data at 17 years and/or 24 years compared to those with incomplete data on these variables.

**Supplementary Table 6.**  Multivariable linear regression models for the relationship between the DQ total z-scores and carotid intima-media thickness and pulse wave velocity at 17 and 24 years, using imputed datasets in the ALSPAC cohort.

**Supplementary Table 6.**  Multivariable linear regression models for the relationship between the DQ scores at 7 years and carotid intima-media thickness and pulse wave velocity at 17 years, using complete case datasets in the ALSPAC cohort.

**Supplementary Table 7.**  Multivariable linear regression models for the relationship between the DQ scores at 10 years and carotid intima-media thickness and pulse wave velocity at 17 years, using complete case datasets in the ALSPAC cohort.

**Supplementary Table 8.**  Multivariable linear regression models for the relationship between the DQ scores at 13 years and carotid intima-media thickness and pulse wave velocity at 17 years, using complete case datasets in the ALSPAC cohort.

**Supplementary Table 9.**  Multivariable linear regression models for the relationship between the DQ scores at 7 years and carotid intima-media thickness and pulse wave velocity at 24 years, using complete case datasets in the ALSPAC cohort.

**Supplementary Table 10.**  Multivariable linear regression models for the relationship between the DQ scores at 10 years and carotid intima-media thickness and pulse wave velocity at 24 years, using complete case datasets in the ALSPAC cohort.

**Supplementary Table 11.**  Multivariable linear regression models for the relationship between the DQ scores at 13 years and carotid intima-media thickness and pulse wave velocity at 24 years, using complete case datasets in the ALSPAC cohort.
